# Skipping Breakfast and Academic Grades, Persistent Feelings of Sadness or Hopelessness, and School Connectedness Among High School Students — Youth Risk Behavior Survey, United States, 2023

**DOI:** 10.15585/mmwr.su7304a10

**Published:** 2024-10-10

**Authors:** Sarah A. Sliwa, Caitlin L. Merlo, Izraelle I. McKinnon, Julie L. Self, Christopher J. Kissler, Ryan Saelee, Catherine N. Rasberry

**Affiliations:** ^1^Division of Adolescent and School Health, National Center for Chronic Disease Prevention and Health Promotion, CDC; ^2^Epidemic Intelligence Service, CDC; ^3^Division of Nutrition Physical Activity and Obesity, National Center for Chronic Disease Prevention and Health Promotion, CDC

## Abstract

Breakfast consumption is positively associated with academic achievement and diet quality among students, whereas skipping breakfast has been linked with poor mental health. Data from CDC’s 2023 nationally representative Youth Risk Behavior Survey were used to describe how often high school students ate breakfast in the past 7 days and the associations between skipping breakfast every day (ate breakfast on 0 of the past 7 days), experiencing persistent feelings of sadness or hopelessness, school connectedness, and self-reported grades. Prevalence estimates and corresponding 95% CIs were calculated, and *t*-tests were used to identify differences within demographic groups (e.g., sex, race and ethnicity, and sexual identity). Logistic regression analyses were conducted to calculate prevalence ratios describing breakfast skipping, adjusting for demographics, and stratified by sex and race and ethnicity. Most students missed breakfast ≥1 time in the past 7 days (72.6%), and 17.9% of students skipped breakfast every day, with differences by sex, sexual identity, and race and ethnicity. Overall, and among both males and females, students who experienced persistent feelings of sadness or hopelessness were more likely to skip breakfast every day. The association between feelings of sadness and hopelessness and skipping breakfast was generally consistent across racial and ethnic groups. In contrast, greater levels of school connectedness and earning mostly As or Bs were inversely associated with skipping breakfast. Students who had higher school connectedness were approximately 30% less likely to skip breakfast on all 7 days. Skipping breakfast and poor mental health co-occur among many adolescents and might impede students’ readiness to learn. School efforts to make breakfast accessible and appealing to high school students might yield multiple benefits and help reinforce school administrators’ efforts to recover student learning losses that occurred during the COVID-19 pandemic. Parents, school decision-makers, and organizations that partner with schools and families can use these findings to guide efforts to promote breakfast consumption.

## Introduction

At the beginning of the 2022–23 school year, many school administrators faced pressure to address declines in students’ mental health and accelerate learning recovery (*1*). Identifying strategies that could support the mental health of students and improve their readiness to learn became paramount. Promoting breakfast consumption can be one such strategy.

Compared with skipping breakfast, consuming breakfast can help support students’ readiness to learn and has been associated with better cognitive performance and academic achievement (*2*) and diet quality (*3*). Healthier dietary patterns, including higher whole grain, fruit, and vegetable intakes, have been positively associated with measures of executive functioning, such as inhibitory control and attention (*4*). A systematic review of longitudinal studies and randomized clinical trials found that, in 10 of 11 studies, eating breakfast more frequently was associated with diet quality improvements in children and adolescents (*5*). Previous analyses of Youth Risk Behavior Survey (YRBS) data have demonstrated that daily breakfast consumption among adolescents has been declining since 2009 (*6*). This is concerning because, beyond its relevance for academic outcomes, breakfast consumption and, more specifically, the lack of breakfast consumption (i.e., skipping breakfast) has been linked to indicators of poor mental health among adolescents (e.g., depression, stress, psychological distress, and anxiety) (*7*).

To help guide schools’ efforts to better support students’ mental health, CDC highlights the relevance of school connectedness, which is associated with better mental health and academic outcomes and can also be protective against a broader set of adverse health behaviors and experiences (*8*). However, the relation between school connectedness and breakfast has not been previously explored, a gap noted in a review about breakfast and the achievement gap among urban, minority youths (*9*). This report estimates the prevalence of breakfast skipping, overall and by demographic characteristics, and describes the association between skipping breakfast and persistent feelings of sadness or hopelessness and school connectedness, both among students overall and stratified by sex and race and ethnicity. Parents, school decision-makers, and organizations that partner with schools and families can use these findings to guide efforts to promote breakfast consumption.

## Methods

### Data Source

This report includes data from the 2023 YRBS (N = 20,103), a cross-sectional, school-based survey conducted biennially since 1991. Each survey year, CDC collects data from a nationally representative sample of public and private school students in grades 9–12 in the 50 U.S. states and the District of Columbia. Additional information about YRBS sampling, data collection, response rates, and processing is available in the overview report of this supplement (*10*). The prevalence estimates for all variables for the study population overall and stratified by demographic characteristics are available at https://nccd.cdc.gov/youthonline/App/Default.aspx. The full YRBS questionnaire, data sets, and documentation are available at https://www.cdc.gov/yrbs/index.html. Institutional review boards at CDC and ICF, the survey contractor, approved the protocol for YRBS. Data collection was conducted consistent with applicable Federal law and CDC policy.*

### Measures

Breakfast frequency was assessed using a single question: “During the past 7 days, on how many days did you eat breakfast?” Skipping breakfast was defined as consuming breakfast on 0 of the past 7 days. Experiencing persistent feelings of sadness or hopelessness, experiencing school connectedness, and reporting receiving mostly As and Bs in school were each assessed using a single question: “During the past 12 months, did you ever feel so sad or hopeless almost every day for 2 weeks or more in a row that you stopped doing some usual activities?”, “Do you agree or disagree that you feel close to people at school?”, and “During the past 12 months, how would you describe your grades in school?” (Table 1). Demographic variables included sex (male and female), race and ethnicity (American Indian or Alaska Native [AI/AN], Asian, Black or African American [Black], Hispanic or Latino [Hispanic], multiracial, Native Hawaiian or other Pacific Islander [NH/OPI], and White), grade (9 and 10 versus 11 and 12), and sexual identity (heterosexual, gay or lesbian, bisexual, questioning [I am not sure about my sexual identity/questioning], or identify in some other way [I describe my identity some other way]). (Persons of Hispanic or Latino origin might be of any race but are categorized as Hispanic; all racial groups are non-Hispanic.)

**TABLE 1:** Question wording and analytic coding for included youth behavior and experience variables — Youth Risk Behavior Survey, United States, 2023

Variable	Question	Response option	Analytic coding
Breakfast consumption	During the past 7 days, on how many days did you eat breakfast?	0 days, 1 day, 2 days, 3 days, 4 days, 5 days, 6 days, or 7 days	0 days, 1 day, 2 days, 3 days, 4 days, 5 days, 6 days, 7 days
Skipping breakfast every day	During the past 7 days, on how many days did you eat breakfast?	0 days, 1 day, 2 days, 3 days, 4 days, 5 days, 6 days, or 7 days	0 days, ≥1days
Experienced persistent feelings of sadness or hopelessness	During the past 12 months, did you ever feel so sad or hopeless almost every day for two weeks or more in a row that you stopped doing some usual activities?	Yes or no	Yes or no
School connectedness	Do you agree or disagree that you feel close to people at school?	Strongly agree, agree, not sure, disagree, or strongly disagree	Strongly agree/agree versus not sure/disagree/ strongly disagree
Mostly As or Bs	During the past 12 months, how would you describe your grades in school?	Mostly A’s, Mostly B's, Mostly C's, Mostly D's, Mostly F's, none of these grades, or not sure	Mostly A's/B's, Mostly C's/D's/F's None of these grades/not sure = missing

### Analysis

The prevalence of students eating breakfast on 0 to 7 days in the 7 days before taking the survey was estimated for the overall sample. In addition, the prevalence of skipping breakfast every day (“breakfast skipping”; eating breakfast on 0 of the past 7 days) was stratified by sex, race and ethnicity, grade, and sexual identity group. All prevalence estimates are weighted and presented with 95% CIs. Pairwise *t*-tests were used to identify demographic differences in prevalence estimates. Prevalence ratios, stratified by sex and race and ethnicity and adjusted for sex, race and ethnicity, sexual identity, and grade, were calculated to estimate associations between skipping breakfast and persistent feelings of sadness or hopelessness, school connectedness, and receiving mostly As and Bs, using a separate model for each measure. Prevalence differences and ratios were calculated using logistic regression with predicted marginals. All prevalence estimates and measures of association used Taylor series linearization. Prevalence estimates with a denominator <30 were considered statistically unreliable and therefore were suppressed (*10*); accordingly, estimates for NH/OPI students are not presented for analyses stratified by race and ethnicity. Prevalence ratio estimates were considered statistically significant if the 95% CI did not cross the null value of 1.0. *T*-tests were considered statistically significant at the p<0.05 level. All analyses were conducted using SAS-callable SUDAAN (version 11.0.4; RTI International) to account for the complex sampling design and weighting.

## Results

Overall, 27.4% of students ate breakfast every day in the past 7 days (Figure). Cumulatively, more than half of students (51.8%) consumed breakfast on 3 or fewer days, and 17.9% of students skipped breakfast every day. Female students had a higher prevalence of skipping breakfast every day than males (19.7% versus 16.2%). Racial and ethnic differences were observed in the percentage who skipped breakfast every day. A larger proportion of Hispanic students (20.0%) reported skipping breakfast every day than White (16.2%) or AI/AN (11.1%) students. Students who identified as heterosexual reported a lower prevalence of skipping breakfast every day (15.8%) than students who identified as gay or lesbian (23.3%), bisexual (25.0%), or questioning (20.7%) (Table 2).

**FIGURE:**
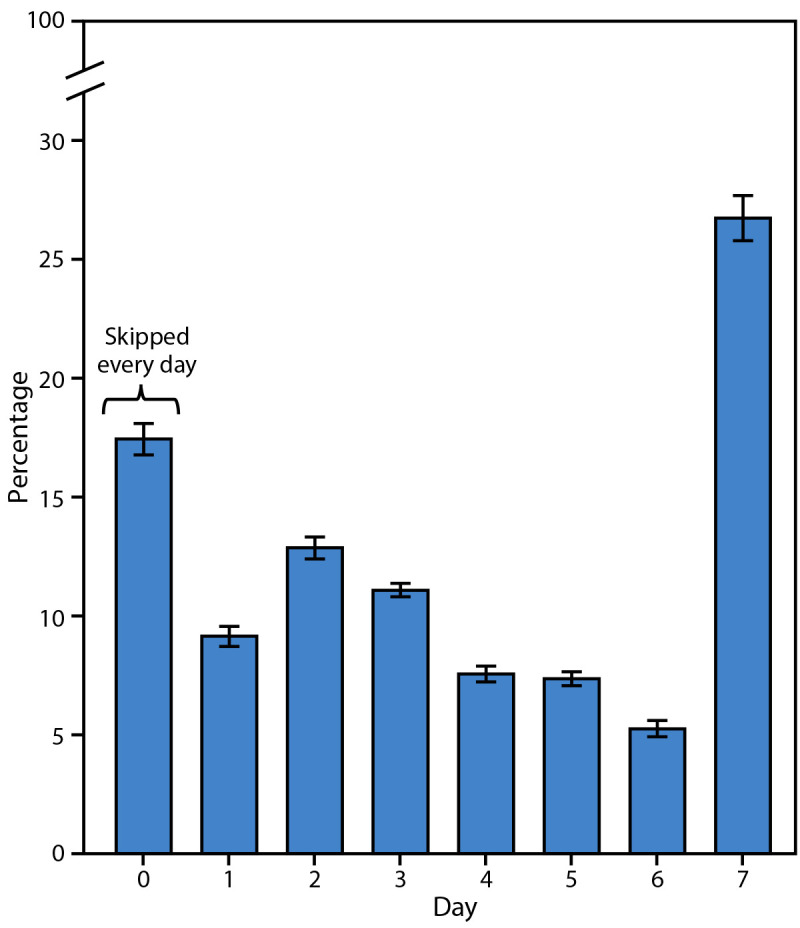
Prevalence of breakfast consumption during the past 7 days among students in grades 9–12, by number of days — Youth Risk Behavior Survey, United States, 2023* *Bars indicate 95% CI.

**TABLE 2:** Percentage of skipping breakfast every day among high school students — Youth Risk Behavior Survey, United States, 2023*

Characteristic	Skipping breakfast (Ate breakfast on 0 of the past 7 days) % (95% CI)
**Sex^†^**	
Female	19.7 (18.1%–21.5%)
Male	16.2 (14.4%–18.1%)
**Race and ethnicity^†,§^**	
American Indian or Alaska Native	11.1 (5.7–20.5)
Asian	15.8 (12.3–20.1)
Black or African American	18.4 (15.7–21.4)
Native Hawaiian or other Pacific Islander	24.0 (14.2–37.7)
White	16.2 (14.7–17.9)
Hispanic or Latino	20.0 (17.4–22.9)
Multiracial	17.9 (14.6–21.7)
**Grade^†^**	
9–10	17.7 (15.9–19.7)
11–12	17.9 (16.5–19.3)
**Sexual identity^†,¶^**	
Heterosexual	15.8 (14.3–17.5)
Gay or Lesbian	23.3 (18.3–29.2)
Bisexual	25.0 (21.2–29.2)
Identify in some other way	19.1 (15.1–23.7)
Questioning	20.7 (16.3–25.9)
**Total**	**17.9 (16.5–19.4)**

* N = 20,103 respondents. The total number of students answering each question varied. Data might be missing because 1) the question did not appear in that student’s questionnaire, 2) the student did not answer the question, or 3) the response was set to missing because of an out-of-range response or logical inconsistency. Percentages in each category are calculated on the known data.

**^†^** Significant difference at p<0.05 calculated using *t*-tests with Taylor series linearization:

Prevalence of skipping breakfast (unadjusted): male <female; American Indian or Alaska Native, White <Hispanic or Latino; heterosexual <gay or lesbian, bisexual, questioning; describe another way <bisexual.

**^§^** Persons of Hispanic or Latino (Hispanic) origin might be of any race but are categorized as Hispanic; all racial groups are non-Hispanic.

**^¶^** Students self-identified their sexual identity with the following response options: heterosexual, gay or lesbian, bisexual, I describe my identity some other way, I am not sure about my sexual identity (questioning), I am not sure what this question is asking (missing).

Experiencing persistent feelings of sadness or hopelessness in the past 12 months was associated with being more likely to skip breakfast every day in the overall sample, as well as among both male and female students and among some, but not all, racial and ethnic groups. Female students who reported persistent feelings of sadness or hopelessness were 64% (adjusted prevalence ratio [aPR] = 1.64) more likely to skip breakfast every day, and males were 37% (aPR = 1.37) more likely to skip breakfast every day compared with those who did not report persistent feelings of sadness or hopelessness in the past year. Prevalence of skipping breakfast every day was higher among students that experienced persistent feelings of sadness or hopelessness for students of most racial and ethnic backgrounds, except among AI/AN and multiracial students (Table 3).

**TABLE 3:** Percentage of feelings of persistent sadness or hopelessness, school connectedness, academic grades, and skipping breakfast among high school students, by sex and race and ethnicity — Youth Risk Behavior Survey, United States, 2023*

Characteristic	Experienced persistent feelings of sadness or hopelessness % (95% CI)	Experienced greater school connectedness % (95% CI)	Reported receiving mostly As or Bs % (95% CI)
**Skipping breakfast** (Ate breakfast on 0 of the past 7 days)			
Overall prevalence	23.2 (21.1–25.4)	15.1 (13.1–17.4)	15.4 (13.7–17.2)
Overall prevalence ratio	1.61 (1.41–1.84)	0.69 (0.59–0.80)	0.64 (0.56–0.73)
**Adjusted prevalence ratios (aPR)** ^†^	**aPR (95% CI)**	**aPR (95% CI)**	**aPR (95% CI)**
Overall^§^	1.51 (1.32–1.73)	0.73 (0.64–0.84)	0.65 (0.56–0.75)
**Sex** ^¶^			
Female	1.64 (1.29–2.07)	0.72 (0.61–0.85)	0.67 (0.53–0.85)
Male	1.37 (1.16–1.62)	0.74 (0.63–0.87)	0.63 (0.54–0.72)
Race and ethnicity**^,††^			
American Indian or Alaska Native	3.47 (0.88–13.78)	0.14 (0.05–0.37)	2.09 (0.56–7.85)
Asian	1.53 (1.05–2.24)	0.59 (0.28–1.25)	0.35 (0.19–0.65)
Black or African American	1.37 (1.06–1.79)	0.63 (0.47–0.85)	0.85 (0.65–1.13)
Native Hawaiian or other Pacific Islander^§§^	—	—	—
White	1.49 (1.22–1.81)	0.77 (0.65–0.93)	0.57 (0.48–0.66)
Hispanic or Latino	1.65 (1.23–2.22)	0.75 (0.53–1.08)	0.76 (0.56–1.03)
Multiracial	1.14 (0.77–1.69)	0.76 (0.45–1.27)	0.51 (0.32–0.84)

* N = 20,103 respondents. The total number of students answering each question varied. Data might be missing because 1) the question did not appear in that student’s questionnaire, 2) the student did not answer the question, or 3) the response was set to missing because of an out-of-range response or logical inconsistency. Percentages in each category are calculated on the known data.

^†^ aPRs were considered statistically significant if the 95% CIs did not cross the null value of 1.0.

^§^ Adjusted for sex, sexual identity, grade, and race and ethnicity.

^¶^ Adjusted for sexual identity, grade, and race and ethnicity.

** Persons of Hispanic or Latino (Hispanic) origin might be of any race but are categorized as Hispanic; all racial groups are non-Hispanic.

^††^ Adjusted for sexual identity, grade, and sex.

^§§^ Values suppressed in cells where N < 30; estimates for Native Hawaiian or other Pacific Islander not shown.

Students who felt connected to school were less likely to skip breakfast every day overall, for female and male students, and AI/AN, Black, and White students. In the overall sample, school connectedness was associated with students being 27% less likely to skip breakfast every day (aPR = 0.73), with estimates for racial and ethnic groups ranging from 23% less likely among White students (aPR = 0.77) to 86% less likely among AI/AN students (aPR = 0.14) (Table 3).

Students who received mostly As or Bs were less likely to skip breakfast everyday overall, among both female and male students, and among students from most racial and ethnic groups, except for among Black and Hispanic students. Female students who received mostly As or Bs were 33% less likely to skip breakfast every day and males were 37% less likely to skip breakfast compared with students who earned mostly Cs, Ds, and Fs. Asian, multiracial, and White students who earned mostly As or Bs were also less likely to skip breakfast every day (Table 3).

## Discussion

During 2023, most high school students were not eating breakfast daily, and approximately one in six students skipped breakfast on all 7 days before taking the survey. Skipping breakfast every day was more prevalent among female students than male students and among Hispanic students than White students. Cross-sectional data from the Cannabis, Obesity, Mental health, Physical Activity, Alcohol, Smoking, and Sedentary behavior (COMPASS) study in Canada found that skipping breakfast was more common among students who were trying to lose weight and who were not involved in sports (*11*), characteristics that were also more common among female students than male students in 2021 YRBS data and might contribute to the observed sex differences in skipping breakfast (*6*). This report is the first analysis of nationally representative data to examine differences in breakfast skipping by sexual identity and found that skipping breakfast every day was more prevalent among lesbian or gay, bisexual, or questioning youths than among their heterosexual counterparts.

Consistent with existing research, the findings in this report indicate that symptoms of poor mental health (i.e., persistent feelings of sadness or hopelessness) were associated with skipping breakfast every day (*7*). Most studies, including this study, examining the association between breakfast consumption and mental health (e.g., anxiety, depressive symptoms, and stress) in adolescents have been cross-sectional (*7*), limiting understanding of potential causal mechanisms. Other research has linked regular breakfast intake with improvements with diet quality (*3*,*5*), which can help support mental health in children and adolescents (*12*).

Many youths are missing the potential benefits of regular breakfast consumption, including youths experiencing persistent feelings of sadness and hopelessness. Although this report is unable to specify how poor mental health and breakfast skipping influence one another, the findings contribute to a body of literature demonstrating that youths who skip breakfast more frequently are also at greater risk for poor mental health (*7*). Together, these studies illustrate the importance of ensuring that students who are struggling with symptoms of poor mental health are prioritized for efforts to decrease breakfast skipping.

In addition, this study examined the relation between school connectedness and breakfast skipping because researchers have 1) explicitly called for better exploration of connectedness and dietary behaviors and 2) have highlighted school connectedness as an important factor for supporting mental health and a wide range of health and academic outcomes (*8*,*9*). Social connectedness, more broadly, and school connectedness, specifically, have been elevated by the U.S. Surgeon General as means of improving population-level physical and emotional well-being. This report also is the first to use national YRBS data to examine the relation between school connectedness and a dietary behavior. Findings suggest that higher levels of school connectedness were associated with being less likely to skip breakfast, addressing a noted gap in the evidence (*9*).

This report found an inverse relation between breakfast skipping and students reporting earning mostly As or Bs, underscoring previously observed associations between breakfast consumption and grades. However, the relation between breakfast skipping, grades, and school connectedness was not explored, which is a topic for future research.

Because of the phrasing of the questions and the cross-sectional nature of data in this study, certain questions of interest cannot be answered. For example, it was not possible to differentiate between eating breakfast at home or at school and, without longitudinal data, it was not possible to assess whether school connectedness promotes breakfast eating or vice versa. Students who experience higher levels of school connectedness might have other characteristics that favor breakfast consumption, such as participating in extracurricular sports or better mental health.

Strategies to increase school connectedness might indirectly support breakfast consumption by increasing students’ wanting to be at school and their attendance, which could lead to eating breakfast at school. Alternatively, a welcoming school breakfast program that fosters social inclusion and belonging might contribute to greater school connectedness. Findings from the COMPASS study also indicated that students with higher levels of school connectedness participated in school breakfast programs more often than those with lower connectedness (*11*). Although neither this analysis nor the analysis from COMPASS can establish causality, the plausibility of both interpretations and the broad benefits of regular breakfast consumption suggest that school efforts to make breakfast appealing and accessible could positively influence multiple outcomes (*3*,*7*,*9*).

Although schools are not the only place where breakfast is consumed, they are of strategic importance. At the household level, socioeconomic and behavioral factors, such as food availability, household income, parental education level, two-parent households, and parental breakfast consumption, are known correlates of adolescent breakfast intake (*13*). These factors are not readily modifiable to increase adolescents’ opportunities to eat breakfast. In contrast, approximately 90,000 schools and institutions already participate in the U.S. Department of Agriculture’s School Breakfast Program (SBP), which makes breakfast available to youths at a paid, reduced, or free cost, depending on household income level. As a result, many schools have an existing infrastructure to build from when designing approaches to support breakfast consumption.

Schools can consider various strategies to reduce skipping breakfast, such as standards-based health education, including teaching students about the benefits of eating breakfast every day, as described in the Food and Nutrition module of CDC’s Health Education Curriculum Analysis Tool. Although health education can influence knowledge and attitudes about breakfast consumption, students need opportunities to apply what they are learning. Participating in SBP might be one way for schools to help address disparities in breakfast consumption and help students to overcome individual and household factors (e.g., timing, logistics, or feeling rushed; low household income; and household food insecurity) that might make it difficult to regularly consume breakfast (*9*,*13*) while providing a balanced meal that meets nutrition standards. The Community Preventive Services Task Force recommends Healthy School Meals for All (https://www.thecommunityguide.org/findings/social-determinants-health-healthy-school-meals-all.html), which makes school meals available at no cost to all students in a qualifying school without asking families to fill out applications, as a strategy to advance health equity. Such universal school meal programs were widely implemented during the emergency phase of the COVID-19 pandemic but became less common once the Federal waivers supporting this flexibility expired during the 2022–23 school year, when these YRBS data were collected. Data from the 2022–23 school year indicate decreased participation in school breakfast and a widening gap between breakfast and lunch participation, with the exception of states that had passed legislation to adopt Healthy School Meals for All or had high uptake of the Community Eligibility Provision (*14*). The Community Eligibility Provision offers a mechanism for higher poverty districts, or individual schools within a district, to provide Healthy School Meals for All. This is relevant because universal school meals are associated with higher SBP participation and attendance and might be an important complement to school activities to narrow disparities in academic outcomes, especially because students in higher poverty school districts experienced greater losses in reading and math achievement during the COVID-19 pandemic, compared with students in lower poverty school districts (*1*).

High schools have successfully increased SBP participation by using alternative breakfast models, such as breakfast in the classroom or grab-and-go breakfasts (*15*), which are designed to give students an opportunity to have breakfast after the school day has begun, removing the need to arrive at school early to eat breakfast and reducing any stigma associated with eating breakfast in the cafeteria (*9*), while minimally disrupting learning time. It is unknown how either alternative or traditional cafeteria-based breakfast programs influence school connectedness among high school students or whether intentional program design could enhance connectedness.

## Limitations

General limitations for the YRBS are available in the overview report of this supplement (*10*). The findings in this report are subject to at least six additional limitations. First, the direction of association between skipping breakfast and the behaviors of interest cannot be assessed because of the cross-sectional nature of the data. Second, behaviors and experiences in these analyses have different recall periods (e.g., past 12 months and past 7 days) and might be subject to different recall bias. Third, a single item that focused on whether students feel close to persons at school was used to estimate school connectedness, which certain studies measure as a multidimensional construct (*8*). Fourth, household income might influence breakfast consumption, self-reported grades, and dietary intake; however, it was not possible to adjust for this potentially confounding variable. Fifth, self-reported grades are a limited measure of academic performance. Finally, the measure of breakfast consumption focused solely on frequency. Without information about the location or composition of breakfast, the role that school breakfast programs or breakfast quality play in the observed associations cannot be estimated.

## Future Directions

A novel finding from this study is that students with higher levels of school connectedness, a known protective factor against poor mental health and risky health behaviors (*8*), were less likely to skip breakfast. The relation between school connectedness and breakfast consumption, including the role of school breakfast programs, merits additional exploration through longitudinal designs that include grades 9–12. States, schools, and districts that implement Healthy School Meals for All programs often evaluate program impact on attendance and academic achievement. A systematic review of the impacts of alternative breakfast models found that few of the included studies featured high school students in the United States. Of those, two reported on academic outcomes and none reported on classroom behavior (*15*). Although the studies were limited in number, the results were promising; both reported significant improvements in attendance after the adoption of alternative breakfast models (*15*). The findings in this report also are consistent with extant literature noting a cross-sectional relation between breakfast consumption and indicators of emotional well-being among adolescents (*7*). Together, these findings point to the relevance of including measures of school connectedness and youth mental health alongside indicators of academic achievement and attendance when evaluating the impact of different school breakfast models and Healthy School Meals for All. In addition, studies or evaluations using designs that can help disentangle co-occurring experiences and identities (e.g., gender and sexual identity) are needed to advance causal inferences and help identify potential causal mechanisms.

## Conclusion

Most students skipped breakfast at least once in the past 7 days and 18% skipped breakfast every day. Students who skipped breakfast every day were less likely to report school connectedness or earning mostly As or Bs and more likely to report symptoms of poor mental health. Families influence adolescents’ breakfast consumption at home by providing breakfast and role modeling eating breakfast; however, this might be logistically or economically infeasible in certain households (*7*). Schools can consider alternative school breakfast models, which can increase participation rates and attendance (*15*). Researchers have an opportunity to advance the field by exploring whether strategies to increase breakfast or school breakfast consumption, such as Healthy School Meals for All and alternative serving models, improve students’ school connectedness, grades, and mental health outcomes and whether these benefits differ by student characteristics (e.g., sex, sexual identity, and race and ethnicity). Parents, school decision-makers, and organizations that partner with schools and families can use these findings to guide efforts to promote breakfast consumption.

## Footnotes

* 45 C.F.R. part 46.114; 21 C.F.R. part 56.114.
